# MicroRNA-125b-5p mimic inhibits acute liver failure

**DOI:** 10.1038/ncomms11916

**Published:** 2016-06-23

**Authors:** Dakai Yang, Qinggong Yuan, Asha Balakrishnan, Heike Bantel, Jan-Henning Klusmann, Michael P. Manns, Michael Ott, Tobias Cantz, Amar Deep Sharma

**Affiliations:** 1 Research Group MicroRNA in Liver Regeneration, Cluster of Excellence REBIRTH, Hannover Medical School, Carl-Neuberg Strasse 1, 30625 Hannover, Germany; 2 Department of Gastroenterology, Hepatology and Endocrinology, Hannover Medical School, Carl-Neuberg Strasse 1, 30625 Hannover, Germany; 3 Twincore Centre for Experimental and Clinical Infection Research, Feodor-Lynen-Strasse 7, 30625 Hannover, Germany; 4 Pediatric Hematology and Oncology, Hannover Medical School, 30625 Hannover, Germany; 5 Translational Hepatology and Stem Cell Biology, Cluster of Excellence REBIRTH, Hannover Medical School, Carl-Neuberg Strasse 1, 30625 Hannover, Germany

## Abstract

The lack of broad-spectrum anti-acute liver failure (ALF) therapeutic agents contributes to ALF-related mortality. MicroRNAs (miRNAs) are suggested to be potent serum biomarkers for ALF, but their functional and therapeutic relevance in ALF are unclear. Here we show an unbiased approach, using two complementary miRNA screens, to identify miRNAs that can attenuate ALF. We identify miR-125b-5p as a regulator of cell death that attenuates paracetamol-induced and FAS-induced toxicity in mouse and human hepatocytes. Importantly, administration of miR-125b-5p mimic in mouse liver prevents injury and improves survival in models of ALF. Functional studies show that miR-125b-5p ameliorates ALF by directly regulating kelch-like ECH-associated protein 1, in turn elevating expression of nuclear factor-E2-related factor 2, a known regulator in ALF. Collectively, our findings establish miR-125b-5p as an important regulator of paracetamol-induced and FAS-induced cell death. Thus, miR-125b-5p mimic may serve as a broad-spectrum therapeutic attenuator of cell death during ALF.

## Abstract (web summary)

miR125b-5p has been associated with acute liver failure. Here the authors show that this miRNA targets Keap1 and activates Nrf2 to inhibit liver cell death after APAP or FAS toxicity, plus they show that a miR125b-5p mimic can inhibit ALF in mice.

## Graphical abstract

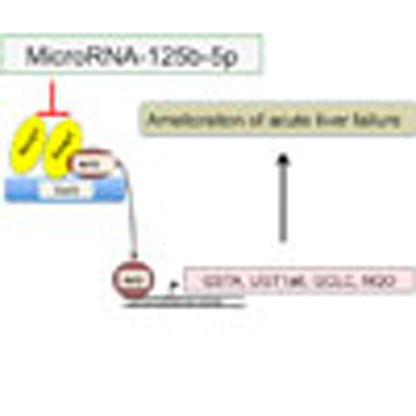

Acute liver failure (ALF) is a rare multi-organ-failure disease that is usually caused by viral hepatitis or drug toxicity^1^,^2^. ALF patients often undergo orthotopic liver transplantation or otherwise die due to shortage of donor livers^3^. One of the major obstacles in treatment of ALF is the lack of both suitable mechanistic biomarkers and broad-spectrum anti-ALF agents^4^. During the last decade, microRNAs (miRNAs) have been suggested as potential biomarkers for various life-threatening diseases, including ALF^5^,^6^,^7^,^8^,^9^,^10^. For example, miR-122, the most abundant miRNA in the liver, has been identified as a biomarker of ALF in mice and humans^5^,^7^,^11^. Similarly, miR-125b-5p has been reported as one of several miRNAs that have elevated expression in cases of paracetamol overdose^10^. Circulating miRNAs in plasma may even be better biomarkers than classical parameters, such as alanine transaminase (ALT) and aspartate transaminase (AST), as miRNAs precede the injury, whereas ALT and AST follow the injury^9^. In contrast to their use as biomarkers, miRNAs are only now beginning to be explored as therapeutic agents^12^,^13^. An important determinant for the use of miRNAs as ALF biomarkers in the clinic is a clear, mechanistic understanding of their functional roles in liver tissue. Furthermore, identification of miRNAs as broad-spectrum anti-ALF agents, which are capable of attenuating cell death, would have the potential to reduce the mortality rate.

## Results

### Functional miRNA screens identify miR-125b-5p as a regulator of ALF

**Figure 1:**
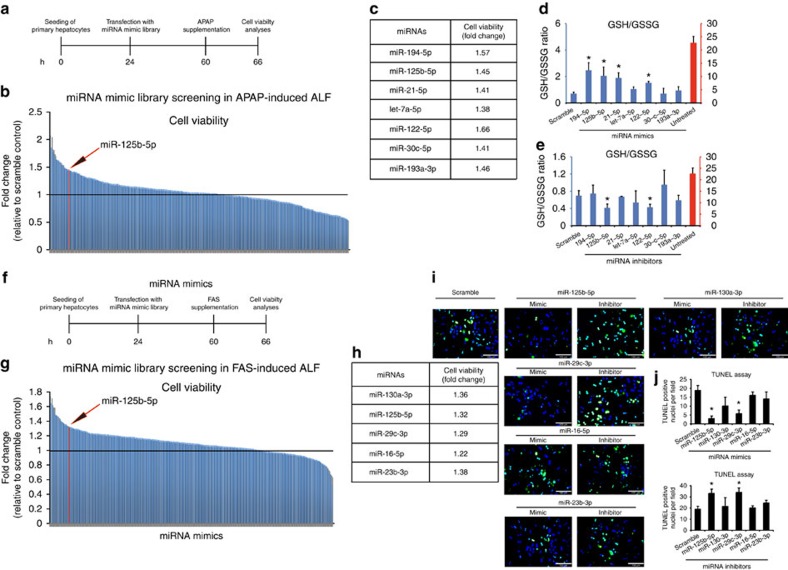
miRNA mimic screening in APAP- or FAS-induced cell death. (**a**) Schematic of miRNA screening in APAP-induced toxicity in primary mouse hepatocytes. (**b**) Cell viability is shown as fold change of each miRNA transfection to scramble control transfection. (**c**) List of miRNAs that are expressed in the liver, either at high or at least moderate levels, and showed more than 20% protection against APAP-induced toxicity compared with scramble control. (**d**,**e**)GSH/GSSG assay of mouse primary hepatocytes transfected with each candidate miRNA mimic (**d**) or inhibitor (**g**), followed by APAP treatment. *P<0.05, one-way analysis of variance (ANOVA). (**f**) Schematic of miRNA screening in FAS-induced hepatocyte toxicity. miRNAs were transfected to primary mouse hepatocytes, followed by treatment of FAS-induced hepatocyte toxicity. (**g**) Cell viability is shown as fold change of each miRNA transfection to scramble control transfection. (**h**) List of miRNAs that are expressed in the liver either at high or at least moderate levels and showed more than 20% protection against FAS-induced hepatocyte toxicity, compared with scramble control. (**i**) Representative photographs (× 200 magnification) of TUNEL assay on mouse primary hepatocytes transfected with each candidate miRNA mimic or inhibitor, followed by FAS treatment. Scale bars, 100 μm. (**j**) Quantification of TUNEL staining shown in **i** *P<0.05, one-way ANOVA. Data are presented as mean±s.e.m. (*n*=4 per group).

We undertook an unbiased screening approach to identify miRNAs that are capable of inhibiting cell death during ALF. We screened for 302 miRNAs from a mimic library, representing the maximum number of completely conserved miRNAs between human and mouse from miRbase version 20.0. First, we functionally screened miRNAs in primary mouse hepatocytes mimicking ALF, induced by paracetamol, also known as acetaminophen or *N*-acetyl-*p*-aminophenol (APAP), the most commonly reported liver toxicity due to drug overdose. Primary hepatocytes were transfected with 25 nM miRNA mimic before onset of APAP-induced toxicity (Fig. 1a). We evaluated all 302 conserved miRNAs and examined cell viability in response to APAP-induced toxicity (Fig. 1b). The hepatoprotective miRNAs were selected based on the following two criteria: (a) an miRNA should provide protection by more than 20% and (b) the miRNA must have high or at least a modest expression in normal human liver^10^. We found seven miRNAs fulfilling these two criteria: miR-194-5p, miR-125b-5p, miR-21-5p, let-7a-5p, miR-122-5p, miR-30c-5p and miR-193a-3p (Fig. 1c). Next, we validated all seven miRNAs in three independent experiments, which confirmed protection against APAP by all seven miRNA mimics, except miR-193a-3p (Supplementary Fig. 1A). We then examined whether inhibition of those seven miRNAs reverses the protective effect against APAP. Inhibition of three miRNAs, let-7a-5p, miR-125b-5p and miR-122-5p, showed lower cell viability compared with control (Supplementary Fig. 1B). Thus, gain- and loss-of-function of let-7a-5p, miR-125b-5p and miR-122-5p inversely influences APAP-induced hepatocyte cell death.

Glutathione (GSH) depletion is one of the hallmarks of APAP-induced hepatotoxicity, which can be detected by measuring the ratio of hepatocyte GSH and glutathione disulfide (GSSG). Hence, to test whether the short-listed seven miRNAs inhibit APAP-induced toxicity specifically, we measured GSH/GSSG ratio in primary hepatocytes transfected with either miRNA mimics or inhibitors. Although we found that mimics of miR-125b-5p, miR-194-5p miR-21-5p and miR-122-5p restored GSH levels significantly (Fig. 1d), inhibitors of only miR-125b-5p and miR-122-5p led to significant reduction in GSH levels (Fig. 1e). Together, miRNA screening and subsequent validation revealed that miR-125b-5p and miR-122-5p protect against APAP-induced hepatocyte toxicity.

Apoptosis is a substantial contributor to ALF, especially in ALF associated with viral hepatitis and acute Wilson's disease^14^,^15^. Therefore, we complemented our APAP-induced ALF miRNA screening with an apoptosis-driven ALF miRNA screening, using a model of FAS/CD95 receptor-induced apoptosis. To induce apoptosis, hepatocytes transfected with miRNA mimics were cultured in the presence of FAS-agonist antibody (anti-CD95 and clone CD95) (Fig. 1f). FAS antibody causes massive apoptosis *in vitro* and *in vivo*, leading to ALF. Likewise, we screened the library of 302 conserved miRNAs in primary mouse hepatocytes, to identify miRNAs that inhibit apoptosis-induced ALF (Fig. 1g). Our screen identified five miRNAs, miR-130a-3p, miR-125b-5p, miR-29c-3p, miR-16-5p and miR-23b-3p, whose mimics suppressed FAS-induced apoptosis in primary hepatocytes (Fig. 1h). The criteria for selection of these five miRNAs were the same as mentioned for the miRNA screening in APAP-induced ALF model. We then validated these five miRNAs in three independent experiments using miRNA mimics and inhibitors. Our cell viability assay showed hepatoprotection by all miRNAs, except miR-16-5p (Supplementary Fig. 2A,B). However, terminal deoxynucleotidyl transferase-mediated dUTP nick-end labelling (TUNEL) assay confirmed hepatoprotection by miR-125b-5p and miR-29c-3p, indicating that only these two miRNAs exhibit the ability to suppress FAS-induced apoptosis (Fig. 1i,j). Based on our complementary miRNA screenings in two different models of ALF, we identified miR-125b-5p as a common miRNA that protected primary hepatocytes *in vitro* against both APAP-induced ALF and FAS-induced ALF. We then tested the dose-dependent effect of miR-125b-5p and found that mimic at the concentration of 25 nM or higher provides significant protection against APAP- and FAS-induced ALF *in vitro* (Supplementary Fig. 3). In addition, we analysed whether APAP influences miR-125b-5p expression and found that miR-125b-5p expression reduces in a dose- and time-dependent manner on ALF induction *in vitro* (Supplementary Fig. 4). Thus, on the basis of our screens and *in vitro* experiments, we selected miR-125b-5p for further studies.

### Administration of miR-125b-5p ameliorates ALF *in vivo*

**Figure 2:**
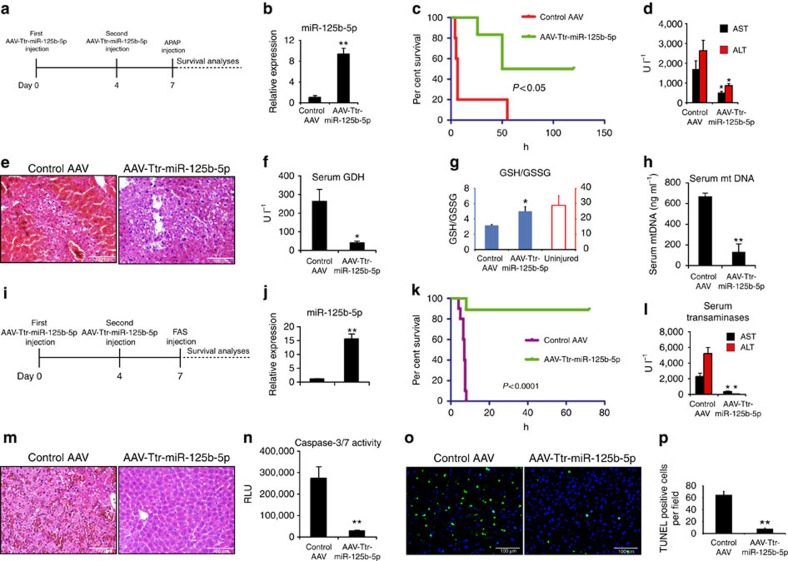
AAV-based miR-125b-5p overexpression inhibits ALF *in vivo*. (**a**) Schematic representation of the experimental design in APAP-induced ALF model. (**b**) Quantitative reverse transcriptase–PCR (qRT–PCR) revealed overexpression of miR-125b-5p in the liver of mice injected with AAV-Ttr-miR-125b-5p compared with liver of mice injected with control AAV. *P<0.05, two-tailed Student's *t*-test. (**c**) Kaplan–Meier survival analyses of mice with or without hepatic miR-125b-5p overexpression (*n*=10 per group) after triggering ALF. *P*<0.05, log-rank test. (**d**) Serum ALT and AST analyses showed less liver damage at 6 h after APAP injection in miR-125b-5p-overexpressing mice. *P<0.05, two-tailed Student's *t*-test. (**e**) Haematoxylin and eosin (HE) staining showed less injury at 6 h after induction of ALF. Scale bars, 100 μm. (**f**) Serum GDH, a mechanistic biomarker (**g**) liver GSH/GSSG ratio and (**h**) serum mtDNA, another mechanistic biomarker, revealed less hepatocyte damage in miR-125b-5p-overexpressing mice after APAP injection. *P<0.05 and **P<0.01, two-tailed Student's *t*-test. (**i**) Schematic representation of the experimental design in FAS-induced ALF model. (**j**) qRT–PCR revealed overexpression of miR-125b-5p in liver of mice injected with AAV-Ttr-miR-125b-5p compared with liver of mice injected with control AAV. **P<0.01, two-tailed Student's *t*-test. (**k**) Kaplan–Meier survival analyses of mice with or without hepatic miR-125b-5p overexpression (*n*=10 per group) after triggering ALF. *P*<0.0001, log-rank test. (**l**) Serum ALT and AST analyses showed less liver damage at 6 h after induction of FAS-induced ALF in miR-125b-5p-overexpressing mice. *P<0.05, two-tailed Student's *t*-test. (**m**) HE staining indicated less apoptosis at 6 h after induction of ALF. Scale bars, 100 μm. (**n**) Caspase-3/7 activity assay showed lower caspase-3/7 activity in mice with miR-125b-5p overexpression, compared with control mice. **P<0.01, two-tailed Student's *t*-test. (**o**) TUNEL assay revealed less cell death in miR-125b-5p-overexpressing mice at 6 h after induction of ALF. Scale bars, 100 μm. (**p**) Quantification of TUNEL staining shown in **o**. **P<0.01, two-tailed Student's *t*-test. Data are presented as mean±s.e.m. from at least three independent experiments.

To examine the therapeutic relevance of miR-125b-5p in ALF, we investigated whether miR-125b-5p protects hepatocytes against APAP-induced ALF in male BALB/c mice (Fig. 2a). At first, we determined an optimal dose by injecting BALB/c mice with various doses of APAP ranging from 150 to 800 mg kg^−1^. We found that administration of 350 mg kg^−1^ APAP or a higher dose leads to 100% lethality in mice. Therefore, we used 350 mg kg^−1^ APAP as a lethal dose in BALB/c mice for further experiments (Supplementary Fig. 5). To overexpress miR-125b-5p in the mouse liver, we cloned pri-miR-125b-5p under the transcriptional control of the hepatocyte-specific promoter transthyretin (Ttr) in an adeno-associated virus (AAV) plasmid and subsequently prepared high-titre AAV serotype 8 encoding miR-125b-5p (henceforth referred to as AAV-Ttr-miR-125b-5p). The successful overexpression of miR-125b-5p was confirmed in BALB/c mice administered with 1 × 10^10^ AAV-Ttr-miR-125b-5p virions via the tail vein (Fig. 2b). We then tested efficacy of miR-125b-5p in an *in vivo* ALF mouse model using BALB/c mice injected with 350 mg kg^−1^, a lethal dose of APAP, intraperitoneally. We observed significantly higher survival in mice injected with AAV-Ttr-miR-125b-5p than in mice injected with a control AAV (Fig. 2c). Surviving mice in AAV-Ttr-miR-125b-5p group were monitored and kept alive for 6 months after the induction of APAP toxicity. Hence, the survival study indicates that miR-125b-5p overexpression in mouse liver renders resistance against APAP-induced ALF and thus improves survival.

Next, we examined whether higher survival observed in mice is indeed due to specific inhibition of APAP toxicity. To address this, a different set of miR-125b-5p-overexpressing mice and control mice was killed 6 h after APAP injection. First, we detected lower levels of alanine transaminase (ALT) and aspartate transaminase (AST) (Fig. 2d), and reduced hepatic injury (Fig. 2e) in miR-125b-5p-overexpressing mice compared with their respective controls. Importantly, decreased serum glutamate dehydrogenase (GDH) levels, elevated GSH/GSSG levels and decreased serum mitochondrial DNA (mtDNA) in miR-125b-5p-overexpressing mice confirmed the specific inhibition of APAP-induced ALF (Fig. 2f–h). It is noteworthy to mention that serum GDH and mtDNA not only confirm ALT and AST values but also represent mechanistic biomarkers for mitochondrial damage as reported previously in mice and humans during APAP-induced liver injury^16^. Thus, miR-125b-5p overexpression improves survival of mice in APAP-induced ALF model.

We then evaluated the protective effect of miR-125b-5p in FAS-induced ALF (Fig. 2i). First, we overexpressed miR-125b-5p in mouse liver by administering AAV-Ttr-miR-125b-5p in BALB/c mice (Fig. 2j) and subsequently injected them with a lethal dose of FAS antibody. Similar to APAP-induced ALF, we observed significantly higher survival of mice injected with AAV-Ttr-miR-125b-5p than their respective controls (Fig. 2k). Furthermore, serum ALT and AST levels, haematoxylin and eosin staining, caspase-3/7 activity assay, TUNEL assay (Fig. 2l–p), cleaved caspase-3 staining and caspase-7 staining (Supplementary Fig. 6A,B) provided evidence that miR-125b-5p overexpression in mice inhibited ALF and hence improved survival.

Notably, a one-time injection of 1 × 10^10^ AAV virions did not exert any effect on the survival of mice. However, when we administered 2 × 10^10^ AAV virions once, we observed improved survival and inhibition of APAP- or FAS-induced ALF (Supplementary Fig. 7). We then investigated whether overexpression of miR-125b-5p influences proliferation in APAP-induced ALF. Our Ki67 staining and quantification revealed the absence of contribution of increased proliferation in APAP-induced ALF (Supplementary Fig. 8A,B). Thus, miR-125b-5p inhibits ALF by suppressing cell death rather than directly effecting proliferation.

**Figure 3:**
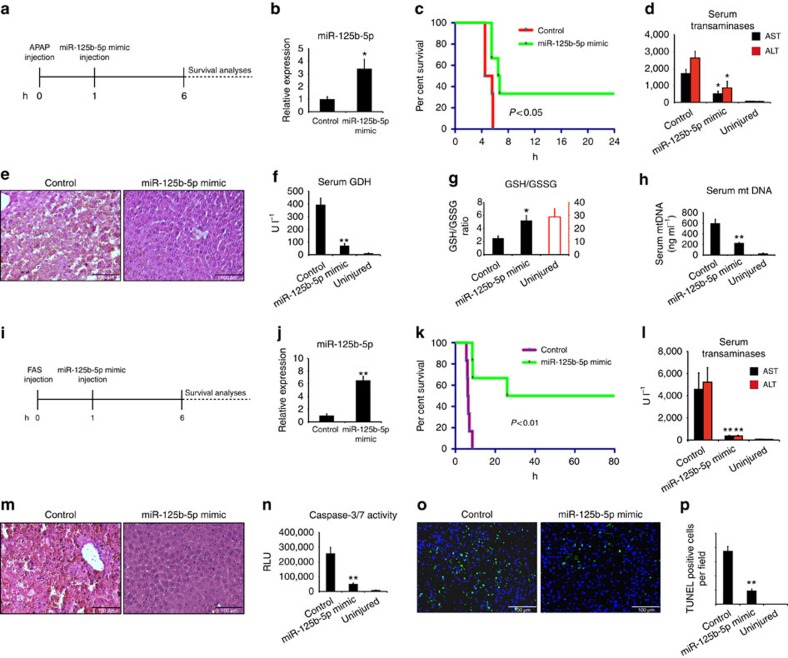
MiR-125b-5p mimic delivery suppresses ALF *in vivo*. (**a**) Schematic representation of the experimental design in APAP-induced ALF model. MiR-125b-5p mimic was injected 1 h after the induction of ALF. (**b**) Quantitative reverse transcriptase–PCR (qRT–PCR) revealed significantly higher levels of miR-125b-5p in the liver of mice injected with miR-125b-5p mimic compared with respective controls. *P<0.05, two-tailed Student's *t*-test. (**c**) Kaplan–Meier survival analyses of mice with or without hepatic miR-125b-5p mimic (*n*=6 per group) after induction of ALF. *P*<0.05, log-rank test. (**d**) Serum ALT and AST analyses showed less liver damage after APAP injection in miR-125b-5p mimic-injected mice. *P<0.05, two-tailed Student's *t*-test. (**e**) Haematoxylin and eosin (HE) staining showed less injury after induction of ALF. Scale bars, 100 μm. (**f**) Serum GDH, a mechanistic biomarker, (**g**) liver GSH/GSSG ratio and (**h**) serum mtDNA, another mechanistic biomarker, revealed less hepatocyte damage in miR-125b-5p mimic-injected mice after APAP injection. **P<0.01, two-tailed Student's *t*-test. (**i**) Schematic representation of the experimental design in FAS-induced ALF model. Similar to APAP model, mice were injected with miR-125b-5p mimic 1 h after the injection of lethal dose of FAS. (**j**) qRT–PCR revealed overexpression of miR-125b-5p in the liver of mice injected with miR-125b-5p mimic compared with respective controls. **P<0.01, two-tailed Student's *t*-test. (**k**) Kaplan–Meier survival analyses of mice with or without hepatic miR-125b-5p mimic (*n*=6 per group) after triggering ALF. It is important to mention that mice that survived in miR-125b-5p mimic-injected group remained alive at later time points as well (monitored for 1 month). *P*<0.05, log-rank test. (**l**) Serum ALT and AST analyses showed less liver damage after induction of FAS-induced ALF in miR-125b-5p mimic-injected mice. **P<0.01, two-tailed Student's *t*-test. (**m**) HE staining indicated less apoptosis after induction of ALF in miR-125b-5p mimic-injected mice. Scale bars, 100 μm. (**n**) Caspase-3/7 activity assay revealed lower caspase-3/7 activity in mice with miR-125b-5p injected mice, compared with control mice. **P<0.01, two-tailed Student's *t*-test. (**o**) TUNEL assay showed less cell death in miR-125b-5p-injected mice after induction of ALF. Scale bars, 100 μm. (**p**) Quantification of TUNEL staining shown in **o**. **P<0.01, two-tailed Student's *t*-test. Data are presented as mean±s.e.m.

AAV-based overexpression in ALF would remain a prophylactic approach; therefore, to test whether miR-125b-5p delivery may serve as a treatment option, we examined the effect of miR-125b-5p in ALF models when injury has already begun. To examine this, we first injected BALB/c mice with 350 mg kg^−1^ APAP. One hour after APAP injection, these mice were administered with stabilized 10 μg miRIDIAN miR-125b-5p mimic (Fig. 3a). We first confirmed the overexpression of miR-125b-5p in mouse liver after administration of miR-125b-5p mimic (Fig. 3b). Our Kaplan–Meier survival curve analyses revealed that miR-125b-5p administration after APAP injection significantly improved the survival of mice (Fig. 3c). Analyses of indicators of APAP-induced ALF revealed reduced ALT and AST levels, reduced hepatic injury, lower serum GDH levels, higher GSH/GSSG ratio and reduced serum mtDNA, suggesting that improved survival was indeed due to inhibition of APAP-induced ALF (Fig. 3d–h). Likewise, we investigated the effect of administration of stabilized 10 μg miRIDIAN miR-125b-5p mimic 1 h after onset of FAS-induced ALF (Fig. 3i). We again confirmed the overexpression of miR-125b-5p expression (Fig. 3j). Importantly, we observed significantly improved survival (Fig. 3k) due to suppressed apoptosis as shown by lower ALT and AST levels, reduced injury, lower caspase-3/7 activity and decreased TUNEL staining in mice injected with stabilized 10 μg miRIDIAN miR-125b-5p mimic (Fig. 3l–p). Thus, these experiments suggest that delivery of miR-125b-5p after onset of ALF is capable of suppressing ALF, and hence leads to improved survival. Therefore, miR-125b-5p mimic delivery may have a therapeutic relevance for treating ALF.

### MiR-125b-5p regulates Keap1 at the post-transcriptional level

**Figure 4:**
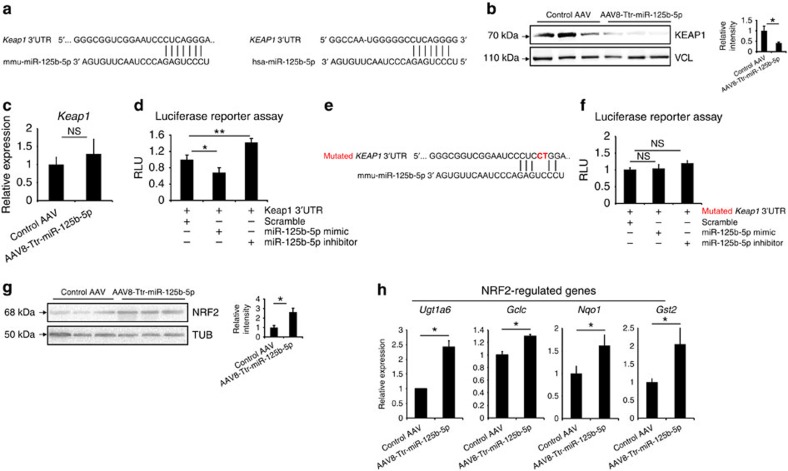
MiR-125b-5p regulates *Keap1* at posttranscriptional level. (**a**) *In silico* analyses by TargetScan and PicTar predicts 3′-UTR of KEAP1 as a target of miR-125b-5p. (**b**) Western blot analysis of KEAP1 in mice treated with AAV-Ttr-miR-125b-5p or control AAV. Vinculin (VCL) was used as a loading control. Quantification of western blotting is shown in the right panel. *P<0.05, two-tailed Student's *t*-test. (**c**) Quantitative reverse transcriptase–PCR (qRT–PCR) analysis shows that *Keap1* mRNA levels did not change significantly. Not significant (NS), two-tailed Student's *t*-test. (**d**) Luciferase reporter assay confirms the binding of miR-125b-5p with 3′-UTR of *Keap1*. Primary hepatocytes transfected with miR-125b-5p mimic have lower relative luciferase units (RLUs), whereas hepatocytes transfected with miR-125b-5p inhibitor showed higher RLUs than the control miRNA scramble transfection. *P<0.05, **P<0.01, one-way analysis of variance (ANOVA). (**e**) Mutated two nucleotides in mouse *Keap1* 3′-UTR are indicated in red. (**f**) Luciferase reporter assay using mutated 3′-UTR of *Keap1* demonstrates unchanged luciferase activity. Not significant (NS), one-way ANOVA. (**g**) Western blot analysis of NRF2 in mice treated with AAV-Ttr-miR-125b-5p or control AAV. α-Tubulin was used as a loading control. Quantification of western blotting is shown in the right panel. *P<0.05, two-tailed Student's *t*-test. (**h**) qRT–PCR analysis of *Ugt1a6*, *Gclc*, *Nqo1* and *Gsta2* expression in mice treated with AAV-Ttr-miR-125b-5p or control AAV. *P<0.05, two-tailed Student's *t*-test. Data are presented as mean±s.e.m. from at least three independent experiments.

The major mechanism by which miRNAs regulate cellular processes is via posttranscriptional regulation by binding to the 3′-untranslated region (3′-UTR) of target messenger RNAs. Therefore, to find a mechanism for the protective effect of miR-125b-5p against ALF, we performed *in-silico* analyses on key regulators of ALF, to find a novel target of miR-125b-5p. We found that miR-125b-5p is predicted to target 3′-UTR of kelch-like ECH-associated protein1 (*Keap*1) (Fig. 4a). Hepatocyte-specific deletion of KEAP1 has been shown to attenuate ALF, indicating that KEAP1 is an important regulator of ALF^17^. Indeed KEAP1 protein levels decreased in miR-125b-5p-overexpressing mice, indicating that miR-125b-5p regulates KEAP1 expression (Fig. 4b and Supplementary Fig. 10a). In contrast, we did not find significant difference in mRNA levels of miR-125b-5p-overexpressing mice and control mice (Fig. 4c). We then investigated whether miR-125b-5p regulates KEAP1 by binding to the 3′-UTR of *Keap*1. To validate predicted binding sites of miR-125b-5p in the 3′-UTR of *Keap1* mRNA, we cloned the 3′-UTR of *Keap1* in a luciferase reporter vector and co-transfected miR-125b-5p and reporter vector in primary mouse hepatocytes. Luciferase reporter assay demonstrated that miR-125b-5p binds to the 3′-UTR and thus regulates KEAP1 directly (Fig. 4d). To further confirm the binding of miR-125b-5p with 3′-UTR of *Keap1* mRNA, we performed the luciferase reporter assay using the mutated 3′-UTR of *Keap1* mRNA (Fig. 4e). Unchanged levels of luciferase activity in the presence of mutated 3′-UTR confirmed that miR-125b-5p regulates the *Keap1* expression at posttranscriptional level (Fig. 4f). Thus, we identified *Keap1* as a novel target of miR-125b-5p.

Next, we examined whether direct regulation of KEAP1 by miR-125b-5p affects subsequent signalling involved in progression of ALF. KEAP1 facilitates degradation of nuclear factor (erythroid-derived 2)-like2 (NRF2) by acting as an adapter for cullin3, a subunit of E3 ubiquitin ligase^18^. KEAP1-NRF2 signalling has been reported to govern key regulatory functions during ALF^17^,^19^. We therefore determined the expression of NRF2 by western blotting. In fact, we found elevated levels of NRF2 in miR-125b-5p-overexpressing mice (Fig. 4g and Supplementary Fig. 10b). Furthermore, the expression of NRF2 target genes such as *Ugt1a6*, *Gclc*, *Nqo1* and *Gsta2* increased in mice injected with AAV-Ttr-miR-125b-5p (Fig. 4h). Thus, miR-125b-5p directly regulates *Keap1*, which leads to enhanced NRF2 signalling and hence inhibits APAP-induced ALF.

**Figure 5:**
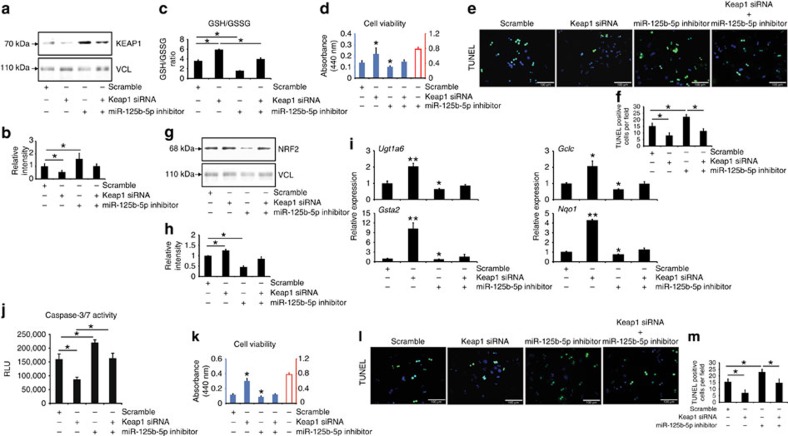
KEAP1 contributes to anti-ALF effects of miR-125b-5p. (**a**) Western blotting shows reduction of KEAP1 protein levels in primary mouse hepatocytes transfected with *Keap1* siRNA. (**b**) Quantification of western blotting shown in **a**. *P<0.05, one-way analysis of variance (ANOVA). (**c**) GSH/GSSG ratio in APAP-treated primary mouse hepatocytes transfected with either *Keap1* siRNA or miR-125b-5p inhibitor alone and together. *P<0.05, one-way ANOVA. (**d**) Cell viability assay of APAP-treated primary mouse hepatocytes transfected with either *Keap1* siRNA or miR-125b-5p inhibitor alone and together. *P<0.05, one-way ANOVA. (**e**) TUNEL assay shows reduced DNA fragmentation in APAP-treated primary mouse hepatocytes transfected with either *Keap1* siRNA or miR-125b-5p inhibitor alone and together. Scale bars, 100 μm. (**f**) Quantification of TUNEL staining shown in **e**. *P<0.05, one-way ANOVA. (**g**) Western blotting for NRF2 and loading control vinculin in primary hepatocytes transfected with Keap1 siRNA or miR-125b-5p inhibitor alone and together. (**h**) Quantification of western blotting shown in **g**. *P<0.05, one-way ANOVA. (**i**) Quantitative reverse transcriptase–PCR (qRT–PCR) analysis of *Ugt1a6*, *Gclc*, *Nqo1* and *Gsta2* expression in primary mouse hepatocytes transfected with either *Keap1* siRNA or miR-125b-5p inhibitor alone and together. *P<0.05, **P<0.01, one-way ANOVA. (**j**) Caspase-3/7 activity assay in FAS-treated primary mouse hepatocytes transfected with either miR-125b-5p inhibitor or *Keap1* siRNA alone and together. *P<0.05, one-way ANOVA. (**k**) Cell viability assay of FAS-treated primary mouse hepatocytes transfected with either *Keap1* siRNA or miR-125b-5p inhibitor alone and together. *P<0.05, one-way ANOVA. (**l**) TUNEL assay performed in FAS-treated primary mouse hepatocytes transfected with either *Keap1* siRNA or miR-125b-5p inhibitor alone and together. Scale bars, 100 μm. (**m**) Quantification of TUNEL staining shown in **l**. *P<0.05, one-way ANOVA. Data are presented as mean±s.e.m. from at least three independent experiments.

A single miRNA often regulates multiple targets simultaneously. Therefore, we asked the question up to what extent does miR-125b-5p exert an anti-ALF effect via KEAP1. To answer this question, we co-transfected primary mouse hepatocytes with miR-125b-5p inhibitor and *Keap1* small interfering RNA (siRNA). We first confirmed the efficacy of *Keap1* siRNA by determining KEAP1 protein levels after transfection of hepatocytes with *Keap1* siRNA alone or in combination with miR-125b-5p inhibitors (Fig. 5a,b and Supplementary Fig. 10c). Importantly, we observed significant inhibition of APAP-induced injury as shown by elevated GSH/GSSG ratio, increased cell viability and reduced TUNEL staining in hepatocytes transfected with siRNA alone compared with respective controls (Fig. 5c–f). Notably, hepatocytes co-transfected with siRNA and miR-125b-5p inhibitors had significantly increased APAP-induced injury compared with hepatocytes that were transfected with *Keap1* siRNA alone (Fig. 5c-f). We further investigated whether suppression of APAP-induced injury via miR-125b-5p affects NRF2 levels and NRF2-responsive genes. Indeed, western blot and quantitative reverse transcriptase—PCR results confirmed that NRF2 protein levels and mRNA levels of *Ugt1a6*, *Gclc*, *Gsta2* and *Nqo1*, respectively, were elevated on transfection of Keap1 siRNA alone or together with miR-125b-5p inhibitor (Fig. 5g–i and Supplementary Fig. 10d).

Likewise, we examined the contribution of KEAP1 in suppression of FAS-induced apoptosis by miR-125b-5p. To this end, we transfected hepatocytes with *Keap1* siRNA alone or in the presence of the miR-125b-5p inhibitor before the induction of apoptosis. Similar to APAP-induced toxicity, we observed inhibition of FAS-induced apoptosis as shown by reduced caspase-3/7 activity, increased cell viability and reduced TUNEL staining in hepatocytes transfected with *Keap1* siRNA alone (Fig. 5j–m). Importantly, hepatocytes co-transfected with the *Keap1* siRNA and miR-125b-5p inhibitor had significantly higher FAS-induced injury compared with hepatocytes transfected with *Keap1* siRNA alone (Fig. 5j–m). It is important to mention that the inhibition of toxicity by *Keap1* siRNA remained less pronounced compared with the miR-125b-5p mimic (Fig. 1). Importantly, hepatocytes co-transfected with miR-125b-5p inhibitor and *Keap1* siRNA showed significantly lower toxicity than hepatocytes transfected with miR-125b-5p inhibitor alone (Fig. 5c–m). Hence, KEAP1 contributes significantly to the anti-ALF effects of miR-125b-5p.

**Figure 6:**
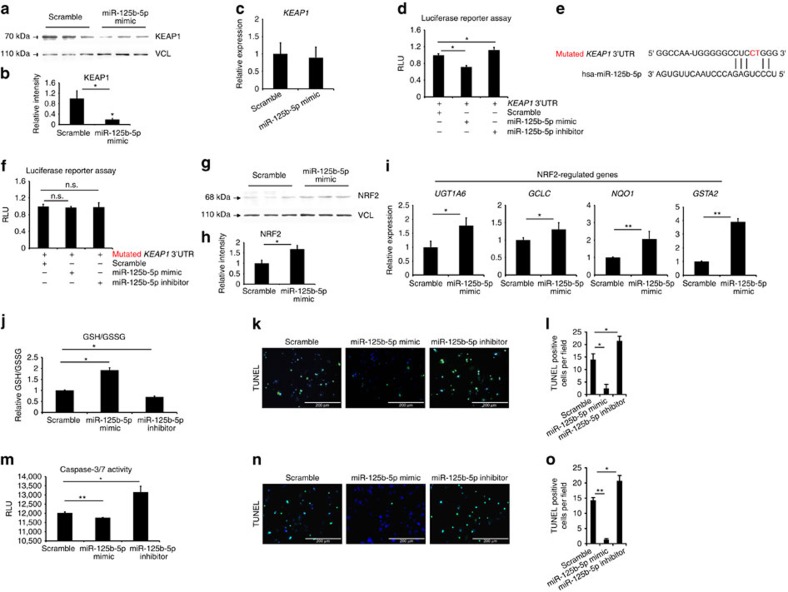
MiR-125b-5p protects human hepatocytes against ALF. (**a**) Western blotting shows reduction of KEAP1 protein levels in primary human hepatocytes transfected with miR-125b-5p mimic. (**b**) Quantification of western blotting shown in **a**. *P<0.05, two-tailed Student's *t*-test. (**c**) Detection of *KEAP1* mRNA levels by quantitative reverse transcriptase–PCR (qRT–PCR) in primary human hepatocytes transfected with miR-125b-5p mimic. (**d**) Luciferase reporter assay confirms the binding of miR-125b-5p with 3′-UTR of human *KEAP1*. Primary human hepatocytes transfected with miR-125b-5p mimic show lower relative luciferase units (RLUs), whereas those transfected with miR-125b-5p inhibitor showed higher RLUs than the scramble miRNA transfection. *P<0.05, one-way analysis of variance (ANOVA). (**e**) Mutated two nucleotides in human *KEAP1* 3′-UTR are indicated in red. (**f**) Luciferase reporter assay using mutated 3′-UTR of *KEAP1* demonstrates unchanged luciferase activity. Not significant (NS), one-way ANOVA. (**g**) Western blot analysis of NRF2 in primary human hepatocytes transfected with miR-125b-5p mimic. Vinculin was used as a loading control. (**h**) Quantification of western blotting is shown in **g**. *P<0.05, two-tailed Student's *t*-test. (**i**) qRT–PCR analysis of *UGT1A6*, *GCLC*, *NQO1* and *GSTA2* expression in primary human hepatocytes transfected with miR-125b-5p mimic or inhibitor. *P<0.05, **P<0.01, two-tailed Student's *t*-test. (**j**) GSH/GSSG assay and (**k**) TUNEL assay revealed reduced hepatocyte damage in miR-125b-5p mimic-treated human hepatocytes after APAP-induced hepatic toxicity. Scale bars, 200 μm. *P<0.05, one-way ANOVA. (**l**) Quantification of TUNEL staining shown in **k**. *P<0.05, one-way ANOVA. (**m**) Caspase-3/7 activity assay and (**n**) TUNEL assay showed less apoptotic cells in miR-125b-5p mimic-treated human hepatocytes after FAS-induced hepatic damage. Scale bars, 200 μm. *P<0.05, **P<0.01, one-way ANOVA. (**o**) Quantification of TUNEL staining shown in **n**. *P<0.05, **P<0.01, one-way ANOVA. Data are presented as mean±s.e.m. from at least three independent experiments.

To test whether miR-125b-5p regulates human KEAP1 as well, we transfected primary human hepatocytes with miR-125b-5p mimic. We observed significant downregulation of KEAP1 levels in miR-125b-5p mimic-transfected primary human hepatocytes (Fig. 6a,b and Supplementary Fig. 10e). KEAP1 mRNA levels remained unchanged, further suggesting posttranscriptional regulation of *KEAP1* by miR-125b-5p (Fig. 6c). We then confirmed direct binding of miR-125b-5p with 3′-UTR of human *KEAP1* but not with mutated 3′-UTR by luciferase assay (Fig. 6d–f), indicating that miR-125b-5p regulates human *KEAP1* at the posttranscriptional level. Subsequently, we showed the miR-125b-5p transfection in primary human hepatocytes leads to significant upregulation of NRF2 protein levels and NRF2-responsive genes such as *UGT1A6*, *GCLC*, *NQO1* and *GSTA2* (Fig. 6g–i and Supplementary Fig. 10f). Thus, miR-125b-5p regulates *KEAP1* and subsequent NRF2 signalling in primary human hepatocytes similar to mouse hepatocytes.

Next, we sought to investigate whether miR-125b-5p can protect human hepatocytes against APAP- and FAS-induced ALF. We transfected miR-125b-5p in primary human hepatocytes that were subsequently exposed to APAP. GSH/GSSG ratio and TUNEL assay revealed that miR-125b-5p alleviates APAP-induced hepatocyte toxicity (Fig. 6k,l). Likewise, transfection of miR-125b-5p inhibited FAS-induced apoptosis in primary human hepatocytes as demonstrated by reduced caspase-3/7 activity and TUNEL assay (Fig. 6m–o). Thus, gain of miR-125b-5p attenuates APAP- and FAS-induced ALF in human hepatocytes as well.

The circulating level of miR-125b-5p has been reported to be elevated in patients with APAP overdose^10^. We therefore measured the levels of miR-125b-5p in sera, isolated hepatocytes and liver of mice injected with 350 mg kg^−1^ APAP. We confirmed the elevated levels of miR-125b-5p in mice sera (Supplementary Fig. 9). In addition, we observed decreased miR-125b-5p levels in hepatocytes and in liver tissue of APAP- or FAS-injected mice (Supplementary Fig. 9). Thus, miR-125b-5p levels follow an inverse correlation between serum and hepatocytes or the liver.

### MiR-125b-5p-KEAP1-NRF2 signaling in ALF patients

**Figure 7:**
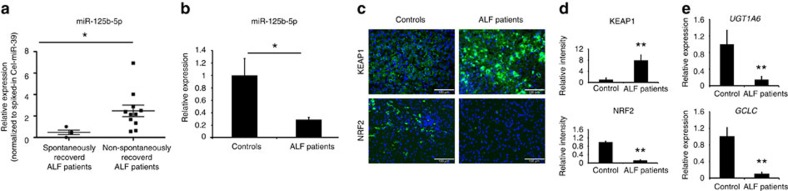
Determination of miR-125b-5p levels in ALF patients. (**a**) Quantitative reverse transcriptase–PCR (qRT–PCR) analyses revealed elevated miR-125b-5p levels in sera of spontaneously recovered compared with non-spontaneously recovered ALF patients. *P<0.05, two-tailed Student's *t*-test. (**b**) Decreased endogenous level of miR-125b-5p in liver biopsies obtained from ALF patients, compared with respective controls. The aetiology of ALF patients (*n*=8) was unknown, except for one patient who had mushroom intoxication. *P<0.05, two-tailed Student's *t*-test. (**c**) Immunofluorescence staining for KEAP1 and NRF2 in liver sections from ALF patients and respective controls. Scale bars, 100 μm. (**d**) ImageJ-based quantification of staining shown in **c**. **P<0.01, two-tailed Student's *t*-test. (**e**) qRT–PCR analysis of *UGT1A6* and *GCLC* in ALF patients and respective controls. **P<0.01, two-tailed Student's *t*-test. Data are presented as mean±s.e.m.

Finally, we addressed whether miR-125b-5p expression is deregulated during ALF in human patients. Upregulation of miR-125b-5p in sera of ALF patients has been recently reported^10^. We also confirmed elevated levels of miR-125b-5p in sera of ALF patients (Fig. 7a). Furthermore, we analysed miR-125b-5p expression in liver biopsies obtained from ALF patients. We found reduced levels of miR-125b-5p in biopsies compared with control livers (Fig. 7b). Consistent with our mouse data, we observed increased protein levels of KEAP1 and reduced protein levels of NRF2 in ALF patients' liver biopsies (Fig. 7c,d). In addition, NRF2-responsive genes such as *UGT1A6* and *GCLC* were expressed at lower levels, thus indicating reduced NRF2 signalling in liver biopsies from ALF patients compared with respective controls (Fig. 7e). Taken together, the loss of hepatic miR-125b-5p expression during ALF, increased levels of serum miR-125b-5p in ALF patients and the alleviation of ALF on miR-125b-5p supplementation in mouse liver indicate that miR-125b-5p is a key regulator of ALF.

## Discussion

The development of novel therapeutic agents to attenuate ALF is needed especially in cases of indeterminate, idiosyncratic and other ALF when *N*-acetylcysteine is ineffective. One of the prerequisites for such an agent is to suppress necrosis and apoptosis, two major cell death modes involved in the progression of ALF. Based on two complementary miRNA screenings in primary hepatocytes, we identified miR-125b-5p as a novel regulator of ALF and uncovered its hepatoprotective function in ALF. Our results demonstrate that miR-125b-5p overexpression attenuates APAP-induced necrosis and FAS-induced apoptosis *in vitro*, as well as ALF, *in vivo*.

Our findings that miR-125b-5p functions as an attenuator of ALF were somewhat unexpected, especially in light of previously reported tumour suppressor functions of miR-125b-5p in liver cancer^20^,^21^. Therefore, our results highlight the fact that an miRNA, such as miR-125b-5p in the liver, may have diverse functions depending on whether liver damage is acute or chronic.

MiR-125b-5p ameliorates APAP-induced ALF via posttranscriptional regulation of *Keap1* mRNA. As a result, NRF2 protein levels are elevated and it translocates to the nucleus to regulate transcription of key genes. Indeed, our results revealed that miR-125b-5p overexpression elevates not only NRF2 levels but also the expression of NRF2-responsive genes such as *Ugt1a6*, *Gclc*, *Nqo1* and *Gsta2*. Therefore, miR-125b-5p regulates GSH levels and hence APAP-induced ALF by modulation of KEAP1-NRF2 signalling. Likewise, apoptosis, which is another mode of cell death, was also inhibited *in vitro* and *in vivo* by anti-apoptotic function of miR-125b-5p via KEAP1-NRF2 signalling. Notably, NRF2 has also been reported to inhibit FAS-induced ALF in mice^22^. On one hand, reduced levels of miR-125b-5p causes NRF2 downregulation via Keap1 signalling but, on the other hand, it is plausible that NRF2 may also contribute to the further changes in miR-125b-5p levels via a feedback loop as reported previously^23^,^24^. It is important to mention that other miR-125b-5p targets in addition to KEAP1 may also contribute to the observed attenuation of ALF, especially in light of our results, demonstrating only partial rescue of the observed effect on blockade of *Keap1* expression using siRNA. Thus, miR-125b-5p acts as an anti-ALF therapeutic agent as demonstrated by our study in two different mouse models of ALF.

In addition to our current findings of protective role of miR-125b-5p during ALF, miR-125b-5p has been previously reported as one of the circulating miRNAs upregulated during the early phase of ALF, at the time when classical markers such as ALT remain low^10^,^25^. We were able to confirm the elevated levels of miR-125b-5p as reported in APAP-overdose patients^10^. The precise mechanism of upregulation of circulating miRNAs in human serum during ALF is currently ambiguous^25^. There are many possibilities that may lead to miR-125b-5p release into serum. MiR-125b-5p may be released from hepatocytes via exosomes or microvesicles or via apoptotic bodies or by random release on cell lysis. It is reasonable to speculate that release via exosomes or microvesicles is the most probable way that can explain miR-125b-5p elevation in serum in the initial stages of ALF^10^. In light of our data demonstrating decrease in hepatic miR-125b-5p levels in biopsies of ALF patients and amelioration of ALF on its overexpression, miR-125b-5p may prove to be a potent ALF regulator and useful mechanistic serum biomarker for the diagnosis and prognosis of ALF.

In summary, based on miRNA screenings, we identified miR-125b-5p as a novel regulator of ALF. Our *in vivo* studies established miR-125b-5p as an anti-ALF miRNA that possesses the capability to inhibit ALF progression. Hence, miR-125b-5p supplementation may serve as therapeutic agent to attenuate ALF.

## Methods

### Mice

All mouse experiments were granted permission and performed according to the guidelines of the Hannover Medical School, Germany. Eight- to 10-week-old male BALB/c mice were purchased from Charles River Laboratories (Germany).

### miRNA mimic library screening

Mouse primary hepatocytes were seeded in 96-well cell culture plates pre-coated with collagen and transfected with mouse miRNA mimic library (Thermo Scientific, Germany) at a final concentration of 25 nM.

### *In vitro* and *in vivo* APAP- and FAS-induced hepatocyte damage

For APAP-induced hepatocyte damage, hepatocytes were treated with 3 mg ml^−1^ APAP (Sigma) at 24 h after miRNA transfection. Likewise, for FAS-induced hepatocyte damage, hepatocytes were treated with 1 μg ml^−1^ Hamster anti-mouse CD95 antibody, clone Jo2 (BD Pharmingen) for (mouse hepatocytes) or mouse anti-human CD95 antibody, clone Dx2 (BD Pharmingen) (for human hepatocytes) at 24 h after miRNA transfection. The cell viability was measured by WST-1 assay (Roche, Switzerland) at 6 h after CD95 or APAP treatment. Absorbance was measured at 440 nm. *In vivo* ALF was induced by intraperitoneal injection of 350 mg kg^−1^ APAP (Sigma) in 8- to 10-week-old BALB/c mice. Likewise, for FAS-induced ALF, 0.5 μg  per gram body weight Hamster anti-mouse CD95 antibody (BD Pharmingen) was injected intraperitoneally. Mice were either monitored for survival or killed 6 h after injection. Liver tissues were harvested and immediately snap frozen in liquid nitrogen and fixed in 4% paraformaldehyde (Sigma).

### Hepatocyte transfection

Freshly isolated primary human hepatocytes were purchased from Cytonet GmBH. Primary hepatocytes from mouse liver were isolated as described^26^. Briefly, mice were anaesthetized and perfused with Liberase (Roche). After perfusion, livers were disintegrated mechanically before collecting hepatocytes by low-speed centrifugation. Non-parenchymal cells were removed by discarding the supernatant. For all *in vitro* transfection experiments, we used Percoll density gradient-purified mouse hepatocytes to achieve high transfection efficiency. Ten thousand primary hepatocytes per well of a collagen-coated 12-well plate (BD) were seeded. Twelve hours after seeding, hepatocytes were transfected with 25 nM miR-125b-5p mimic, miR-125b-5p inhibitor or control scramble (Qiagen), using the Targefect reagent in the presence of virofect enhancer (Targeting Systems). Transfected hepatocytes were cultured in Hepatocyte Culture Medium (Lonza).

### Immunofluorescence and immunohistochemical staining

Hepatocytes were fixed in 4% paraformaldehyde for 15 min at room temperature. Cleaved caspase-3 (Cell Signaling, catalogue number: 9661, 1:400 dilution), cleaved caspase-7 (Cell Signaling: catalogue number: 8438, 1:400 dilution) and Ki67 (Labvision, catatalogue number: RM9106, 1:400 dilution) staining were performed on 10 μm cryosections from mouse liver tissue. AlexaFluor-conjugated secondary antibodies were used for signal detection. For TUNEL staining, a TUNEL assay kit (Merck Millipore) was used according to the manufacturer's guidelines. For haematoxylin and eosin staining, liver tissues were fixed with 4% formalin, embedded in paraffin and cut into 5-μm-thick sections for histochemical analysis.

### Serum parameter analysis

To analyse serum ALT and AST, 0.1 ml blood was collected from each mouse. After 30 min of incubation at room temperature, serum was prepared by centrifuging the samples at 8,000 *g* for 8 min. The clear supernatant was collected and sent to a routine clinical lab for measuring ALT and AST by fully automated Olympus AU 400 analyser (Beckman Coulter, Inc.).

### Gene expression analyses

For gene expression analysis, 1,000 ng total RNA was used for first-strand complementary DNA synthesis (Applied Biosystems). For miRNA expression, 50 ng total RNA was used for miR-cDNA synthesis (Taqman miRNA RT kit, Applied Biosystems). Taqman Universal Real Time PCR kit and SYBR green PCR master mix were purchased from Applied Biosystems. Primers for mouse *Ugt1a6*, *Gclc*, *Nqo1* and *Gsta2*, and β-actin (*Actb*) were purchased from Qiagen. Gene expression was normalized to *Actb*. miRNA expression was normalized to U6. Data were analysed according to the ΔΔCt method.

Mouse cytochrome c oxidase subunit III forward:

5′-ACCAAGGCCACCACACTCCT-3′.

Mouse cytochrome c oxidase subunit III reverse:

5′-ACGCTCAGAAGAATCCTGCAAAGAA-3′.

### AAV serotype 8 preparation

AAV8-Ttr-miR-125b-5p vector and control vector (AAV8-Ttr-Cre) were prepared as described^27^,^28^. Briefly, A-293 cells were transfected with transgene plasmid and pDP8.ape (Plasmid Factory) using calcium-phopsphate transfection method. Three days after transfection, cells were harvested and virus was purified using caesium chloride density gradient centrifugation. The titre was determined by quantitative reverse transcriptase–PCR using primers spanning the region of the Ttr promoter as published before^27^ using the following primers:

Ttr forward primer: 5′-AGCTTGGCAGGGATCAG-3′.

Ttr reverse primer: 5′-GCTTCTCCTGGTGAAG-3′.

### MiR-125b-5p mimic administration *in vivo*

For rapid gain of miR-125b-5p function we injected 10 μg HPLC-purified miRIDIAN miR-125b-5p mimic (Dharmacon) via the tail vein in BALB/c mice that were injected 1 h earlier with APAP or FAS intraperitoneally. The miR-125b-5p dilution and injection was performed according to instructions provided by the manufacturer.

### Immunoblotting

Immunoblottings were performed from whole-cell lysates or mouse liver lysates obtained using Cell Lysis Buffer (Cell Signaling) in combination of Complete Mini Protease Inhibitor Cocktail (Roche) and HALT-phosphatase inhibitor (Thermo Scientific). Protein (200 ng) per sample (BCA Protein Assay Kit, Pierce, USA) was used for western blottings. The antibodies KEAP1 (Abcam, catalogue number: 66620, 1:500 dilution), NRF2 (Abcam, catalogue number: 31163, 1:500 dilution), α-tubulin (Sigma-Aldrich, catalogue number: T9026, 1:1,000 dilution) and vinculin (Sigma-Aldrich, catalogue number: V9131, 1:5,000 dilution) were used. Proteins were visualized using ECL (Thermo Scientific).

### Luciferase reporter assay

The 3′-UTR of *Keap1* mRNA was amplified by PCR from genomic DNA using the following primers:

Mouse *Keap1* forward primer:

5′-GGAAAGTTTAAACGAGAAGCCTCTGGGCTCTG-3′.

Mouse *Keap1* reverse primer:

5′-GGAAATCTAGACCATCAGGATCTGCGTGTATT-3′.

Human *KEAP1* forward primer:

5′-GGAAAGTTTAAACCGGCAGCTGTCACCATGT-3′.

Human *KEAP1* reverse primer:

5′-GGAAATCTAGAACAAAATAACTGTCCATCCGGT-3′.

The amplicon was cloned into a miRGLO vector (Promega). Luciferase reporter assay was performed as described^27^. Briefly, primary hepatocytes were co-transfected with 100 ng miRGLO plasmid containing the 3′-UTR and 25 nM mimic or inhibitor. Forty-eight hours later, Dual-GLO luciferase reagent was first added and incubated for 30 min before measuring the firefly luminescence. StopGLO reagent was then added to cells and incubated again for 30 min before reading the luminescence intensity. Mutated 3′-UTRs were amplified and plasmids were constructed using the QuikChange lightning site-directed mutagenesis kit (Agilent Technologies).

### Caspase 3/7 activity assay

Caspase 3/7 activities in liver tissue were measured using a Caspase-Glo assay kit (Promega)^29^. Briefly, mouse liver lysates were prepared by Dounce homogenization in hypotonic extraction buffer (25 mM HEPES pH 7.5, 5 mM MgCl_2_ and 1 mM EGTA) in combination of Complete Mini Protease Inhibitor Cocktail (Roche) and subsequently centrifuged (15 min, 13,000 r.p.m., 4 °C). One microgram of protein per sample (BCA Protein Assay Kit, Pierce) was used for caspase3/7 activity assay. The luminescence of each sample was measured in a white 96-well plate.

### GDH detection

GDH in mouse serum was detected using Glutamate Dehydrogenase Detection Kit (Abcam), according to manufacturer's recommendation. Briefly, 5 μl serum samples were diluted in assay buffer and mixed with Reaction Mix. The GDH activity was quantified colorimetrically (*λ*=450 nm).

### GSH/GSSG-Glo assay

GSH/GSSG ratio in liver tissue was measured using GSH/GSSG-Glo assay kit (Promega) as per the manufacturer's recommendation. Briefly, mouse liver lysates were homogenized in 5% w/v metaphosphoric acid (Sigma) and subsequently centrifuged (10 min, 13,000 r.p.m., 4 °C). Supernatants were neutralized with neutralization buffer and diluted in dilution buffer. Total GSH and oxidized GSH were measured using a luminescence reader.

### Serum mtDNA measurement

Serum mtDNA was measured by absolute quantification real-time PCR, as described^30^. Briefly, total DNA was isolated from serum samples using a QIAamp Blood and Mini Kit (Qiagen). To construct standard curves, mitochondrial pellets were isolated from mouse liver using Mitochondrial DNA Isolation Kit (Abcam). Purity of mtDNA standards was verified by real-time PCR for mouse cytochrome *c* oxidase subunit III and β-actin.

### Human patient samples

Frozen liver tissues from human ALF patients were obtained from the Department of Gastroenterology, Hepatology and Endocrinology, Hannover Medical School, where all human samples (blood and tissues) were collected after informed consent from patients. The study was performed according to the guidelines of, and given permission by, the ethics committee of Hannover Medical School.

### Serum miRNA analyses

miRNAs were isolated from 100 μl of human serum using the miRNeasy Serum/Plasma Kit (Qiagen) according to the manufacturer's instructions. Serum RNA (2.25 ng) was reverse transcribed using the Taqman miRNA RT kit (Applied Biosystems) and miR-125b-5p expression was determined by real-time PCR using Taqman Universal Real Time PCR kit (Applied Biosystems). miRNA expression was normalized to spike-in control of *Caenorhabditis elegans* miR-39 miRNA. Data were analysed according to the ΔΔCt method.

### Statistical analysis

Significance was determined with the two-tailed Student's *t*-test for comparison of two groups. Significance between multiple groups was determined by one-way analysis of variance. A *P*-value of <0.05 was considered significant. Error bars represent±s.e.m. *P<0.05 and **P<0.01.

### Data availability

The authors declare that data supporting the findings of this study are available within the article and its Supplementary Information files.

## Additional information

**How to cite this article**: Yang, D. *et al*. MicroRNA-125b-5p mimic inhibits acute liver failure. *Nat. Commun.* 7:11916 doi: 10.1038/ncomms11916 (2016).

## Supplementary Material

Supplementary Information Supplementary Figures 1-10.
