# Supplementary material for: MicroRNA-125b-5p mimic inhibits acute liver failure
Source: Nat Commun. 2016 Jun 23;7:11916. doi: 10.1038/ncomms11916 (PMC4931005; doi:10.1038/ncomms11916)
Supplement: Supplementary Information — Supplementary Figures 1-10. [file ncomms11916-s1.pdf]

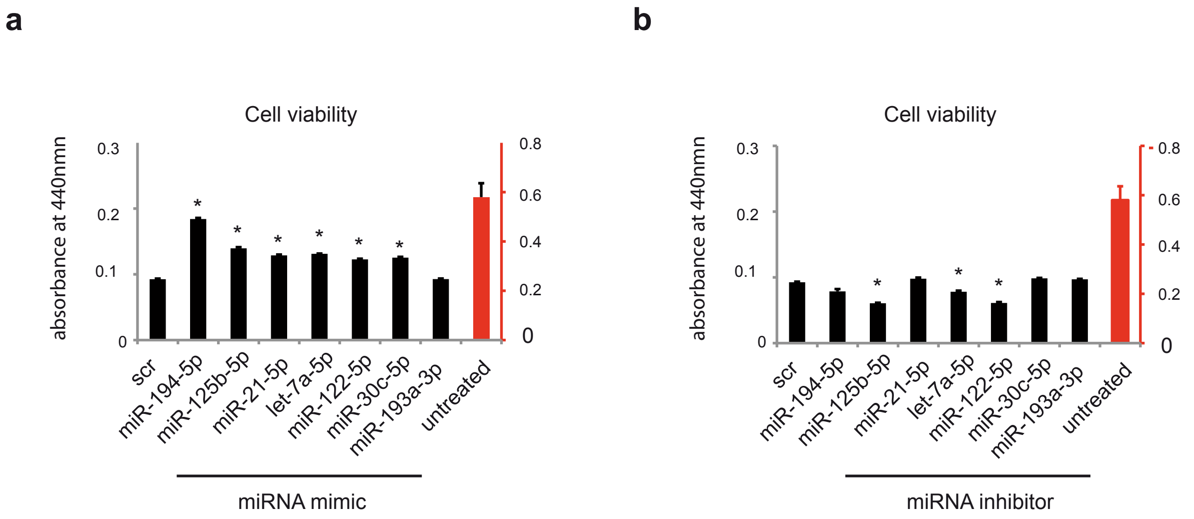

Supplementary figure 1: Validation of miRNA screening in APAP-induced ALF.
(a, b) Validation of miRNA screening by cell viability assay in primary mouse hepatocytes transfected with 25nM of each candidate miRNA mimic (a) or inhibitor (b), followed by APAP treatment. *P<0.05, one-way ANOVA. Data are presented as mean ± SEM (n=4 per group).

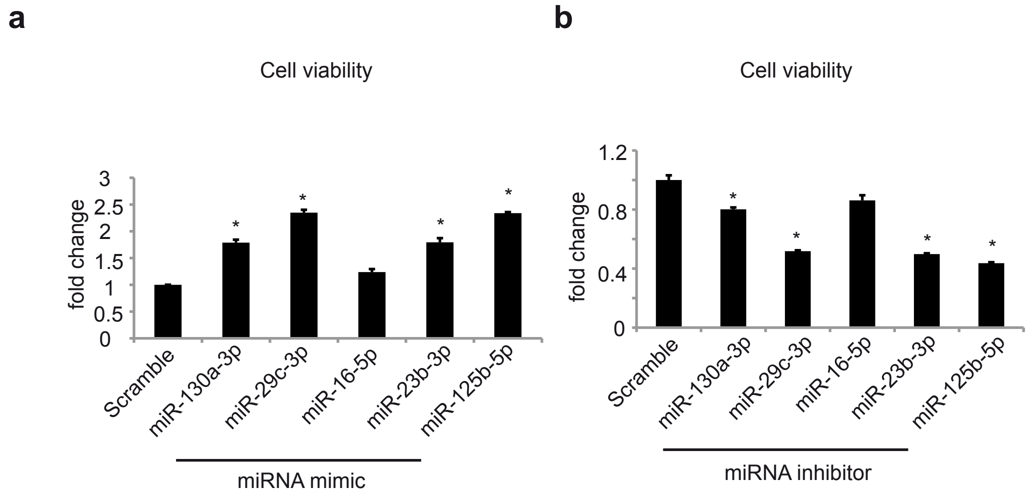

Supplementary figure 2: Validation of miRNA screening in FAS-induced ALF.
(a, b) Cell viability assay of mouse primary hepatocytes transfected with 25nM of each candidate miRNA mimic (a) or inhibitor (b), followed by FAS-induced hepatocyte toxicity. *P<0.05, one-way ANOVA. Data are presented as mean ± SEM (n=4 per group).

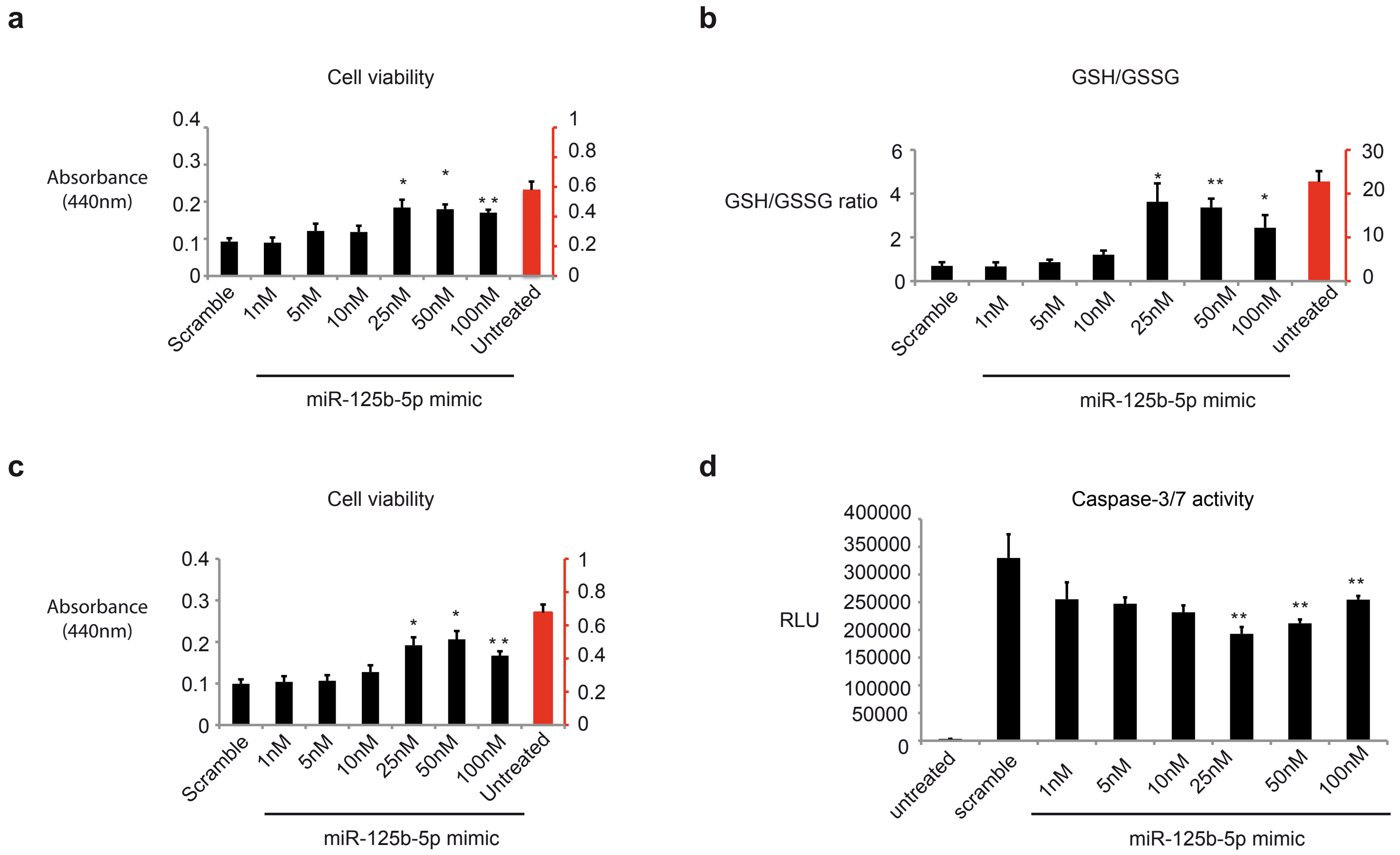

Supplementary figure 3: Dose dependent effects of miR-125b-5p mimic on ALF in vitro.
(a) Cell viability assay and **(b)** GSH/GSSG ratio analyses of APAP-treated primary mouse hepatocytes transfected with indicated concentrations of miR-125b-5p mimic. *P<0.05, **P<0.01, one-way ANOVA. **(c)** Cell viability assay and **(d)** caspase-3/7 activity analyses of FAS-treated primary mouse hepatocytes transfected with indicated concentrations of miR-125b-5p mimic. *P<0.05, **P<0.01, one-way ANOVA. Data are presented as mean ± SEM (n=4 per group).

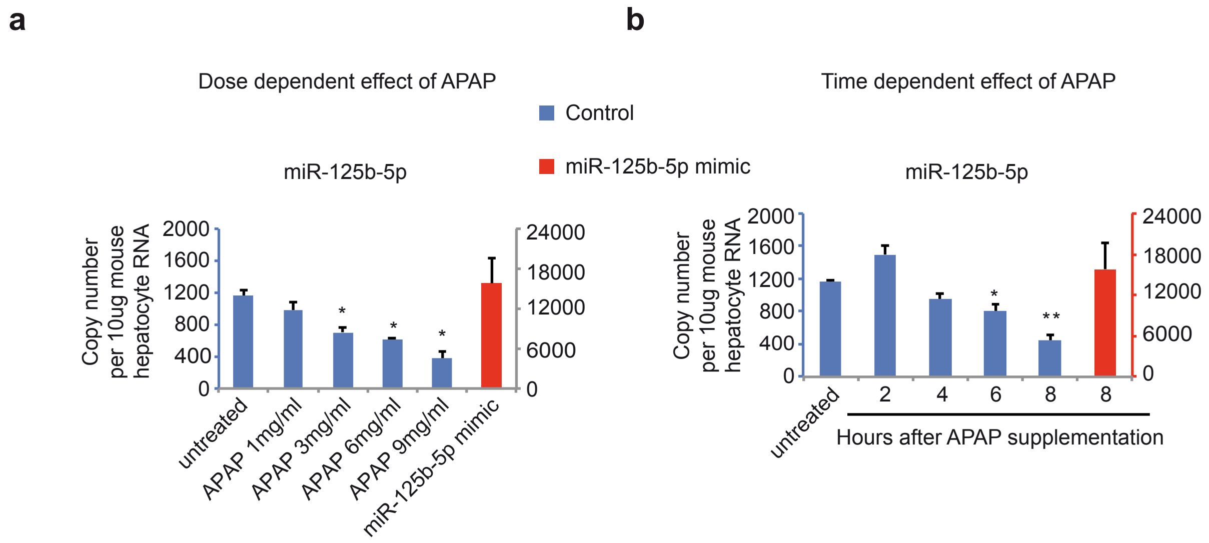

Supplementary figure 4: Dose and time dependent effect of APAP on miR-125b-5p expression. **(a)** Determining the miR-125b-5p levels by qRT-PCR in response to different dose of APAP ranging from 1mg/ml to 9mg/ml. *P<0.05, one-way ANOVA. **(b)** The miR-125b-5p levels were determined at indicated time points by qRT-PCR in primary mouse hepatocytes treated with 3mg/ml APAP. *P<0.05, **P<0.01, one-way ANOVA. Data are presented as mean ± SEM (n=3 per group).

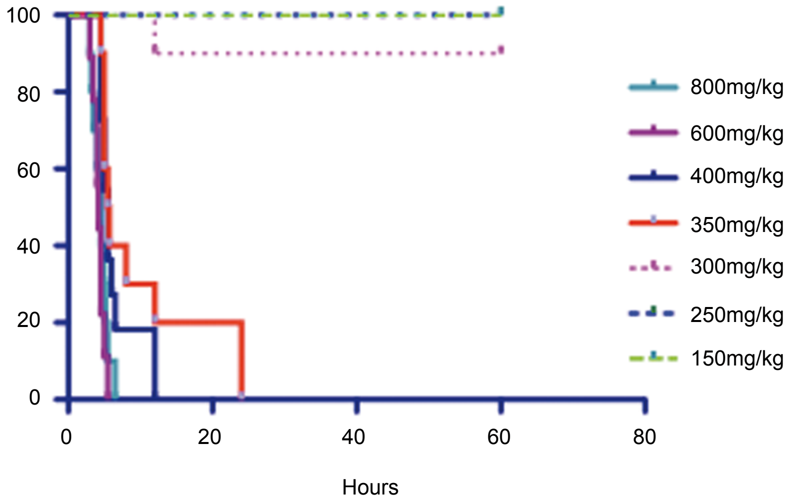

Supplementary figure 5: Determining a lethal dose of APAP in BALB/c mice.
Male BALB/c mice were injected with different dose of APAP ranging from 150mg/kg to 800mg/kg and survival of mice was analyzed by Kaplan-Meier curve (n=10 mice per group).

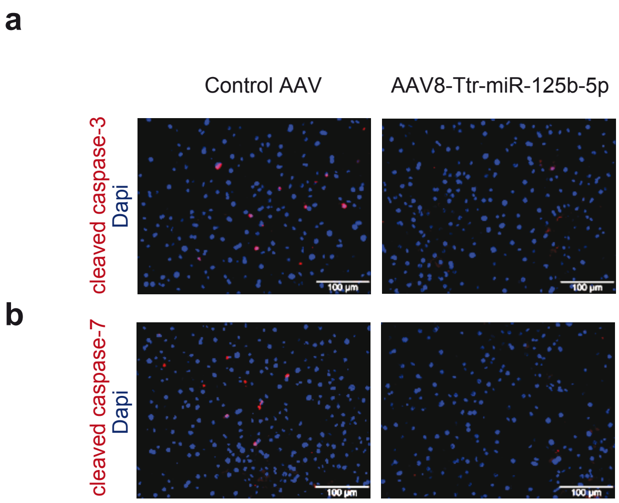

Supplementary figure 6: MiR-125b-5p overexpression inhibits FAS-induced ALF.
(a) cleaved caspase-3 and **(b)** cleaved caspase-7 immunofluorescence staining showed less apoptosis in miR-125b-5p overexpressing mice at 6 hours after induction of ALF. Scale bars, 100μm.

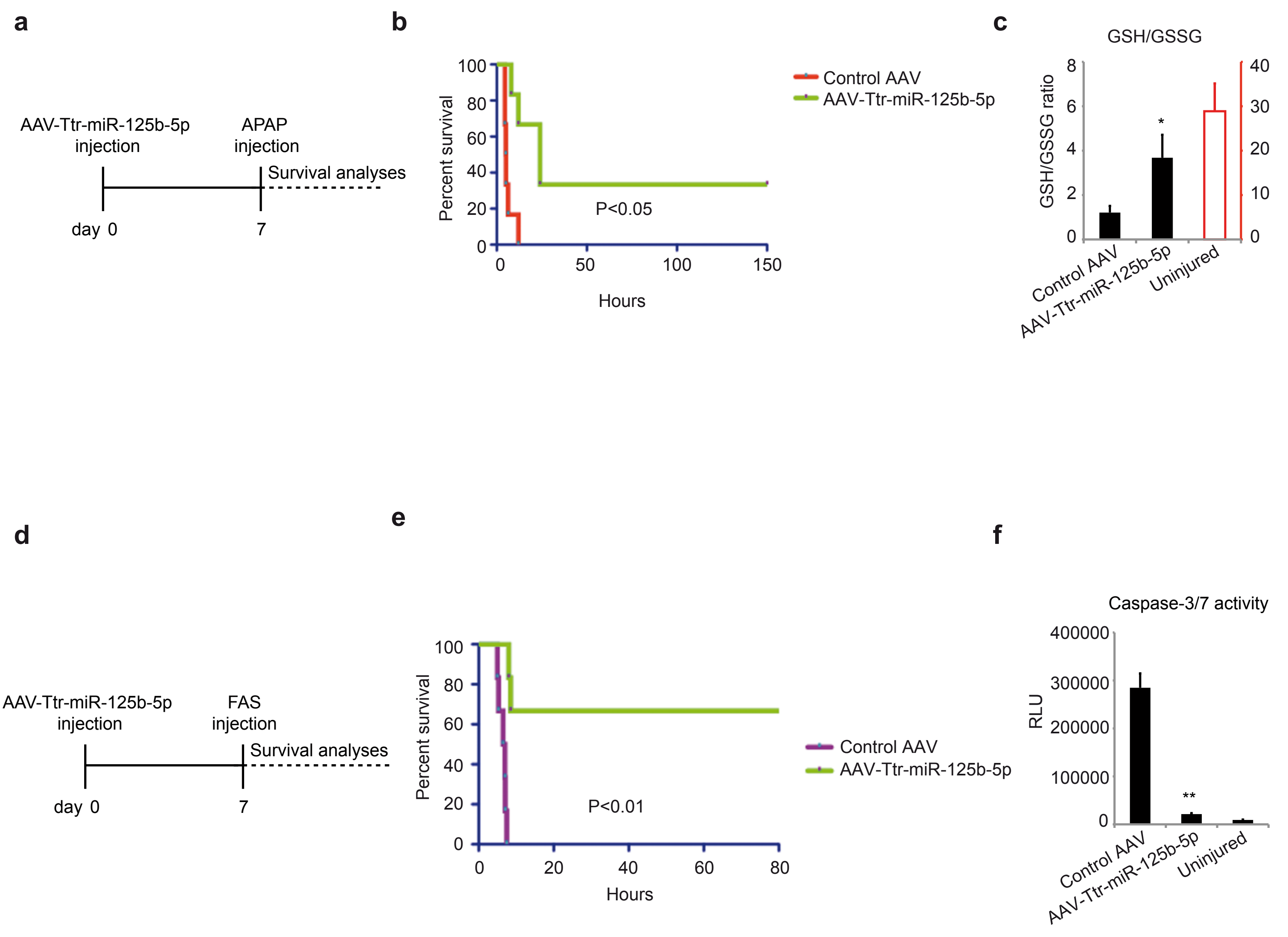

Supplementary figure 7: Administration of 2x10(10) AAV-Ttr-miR-125b-5p only inhibits ALF.
(a) Schematic representation of the experimental design in APAP-induced ALF model. Mice were injected with 2x10(10) AAV-Ttr-miR-125b-5p only once or control AAV seven days prior to APAP injection **(b)** Kaplan-Meier survival analyses of APAP-treated mice injected with 2x10(10) AAV-Ttr-miR-125b-5p or control AAV (n=6 per group). P<0.05, log-rank test. **(c)** Liver GSH/GSSG ratio revealed less hepatocyte damage in APAP-injected mice that were administered with 2x10(10) AAV-Ttr-miR-125b-5p compared to respective controls. *P<0.05, two-tailed Student's t-test. **(d)** Schematic representation of the experimental design in FAS-induced ALF model. Similar to APAP model, mice were injected with 2x10(10) AAV-Ttr-miR-125b-5p only once or control AAV seven days prior to FAS injection. **(e)** Kaplan Meier survival analyses of FAS-treated mice injected with 2x10(10) AAV-Ttr-miR-125b-5p or control AAV (n=6 per group). P<0.01, log-rank test. **(f)** Caspase-3/7 activity assay showed lower caspase-3/7 activity in FAS-injected mice that were administered with 2x10(10) AAV-Ttr-miR-125b-5p compared to control mice. **P<0.01, two-tailed Student's t-test.
Data are presented as mean ± SEM.

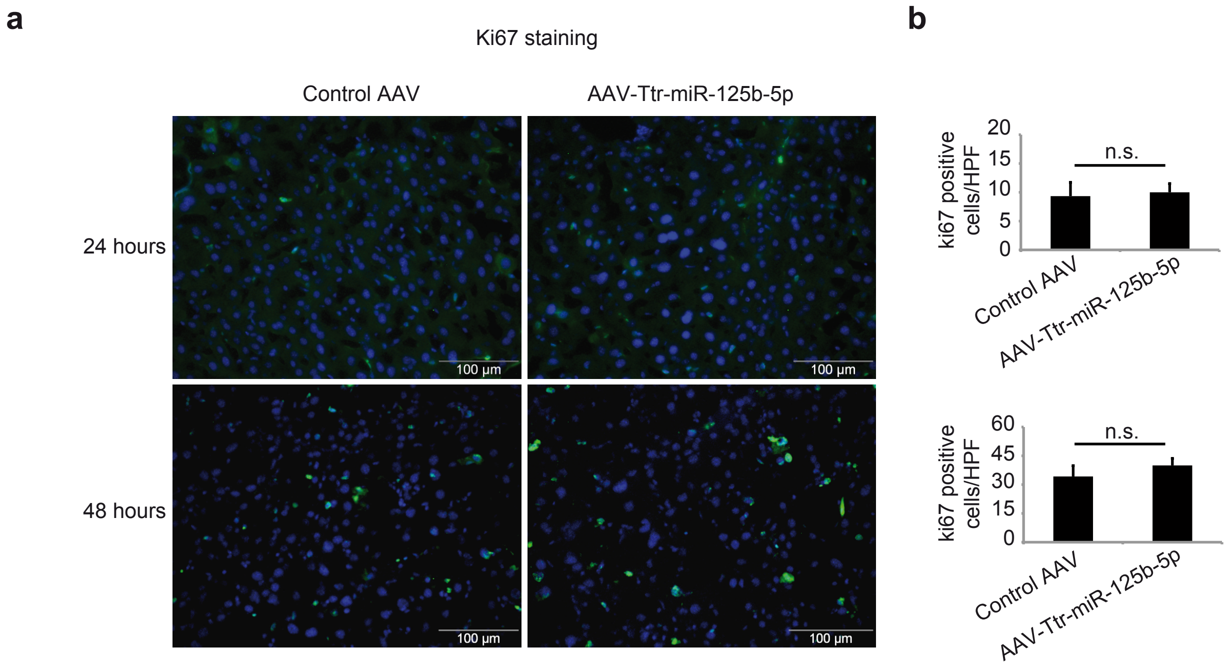

Supplementary figure 8: Analyses of proliferation in APAP-induced model.
(a) Ki67 immunofluorescence staining in liver sections of 250mg/kg APAP-injected mice administered either with AAV-Ttr-miR-125b-5p or control AAV. **(b)** Image-J based quantification of Ki67 staining shown in panel (a). Not significant (n.s.), two-tailed Student's t-test. Data are presented as mean ± SEM.

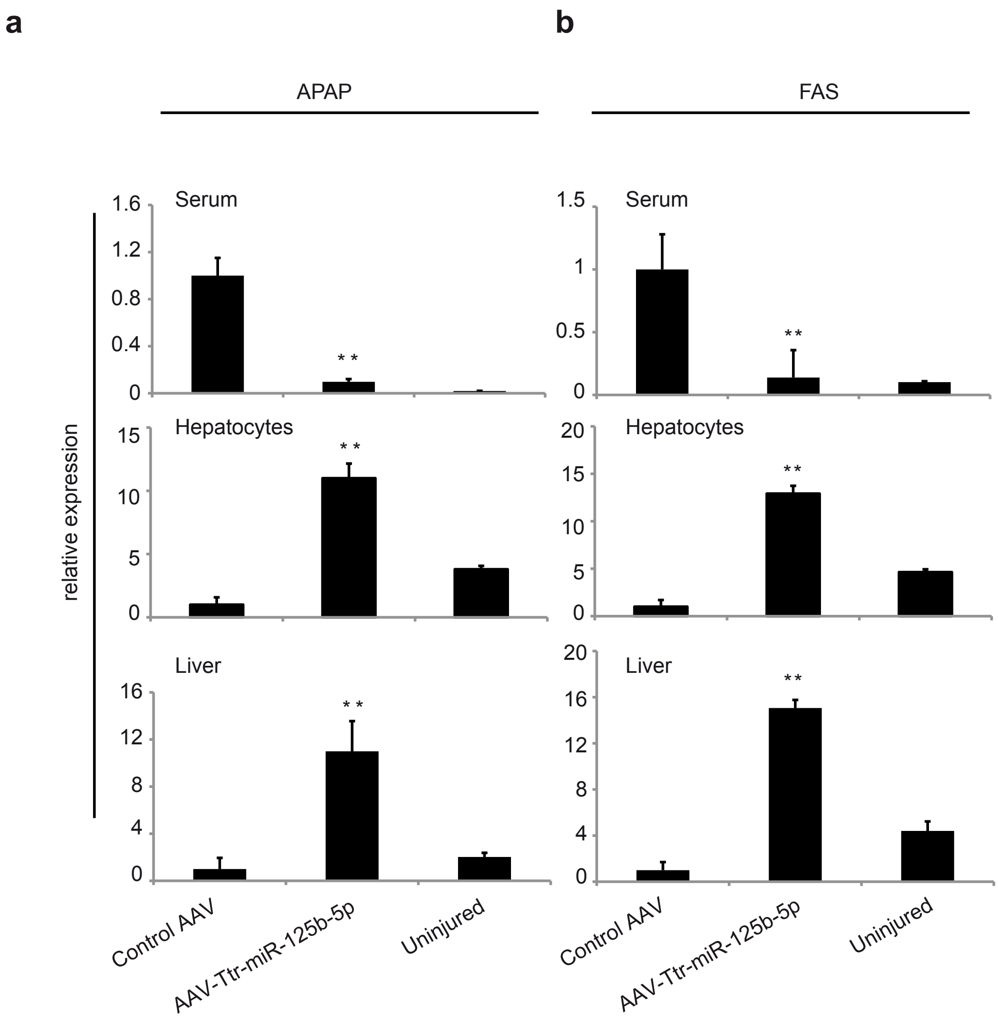

Supplementary figure 9: MiR-125b-5p expression analyses in serum, hepatocytes and liver.
(a) Analyses of miR-125b-5p levels in serum, hepatocytes and liver of same mice treated with APAP or **(b)** with FAS. **P<0.01, two-tailed Student's t-test.

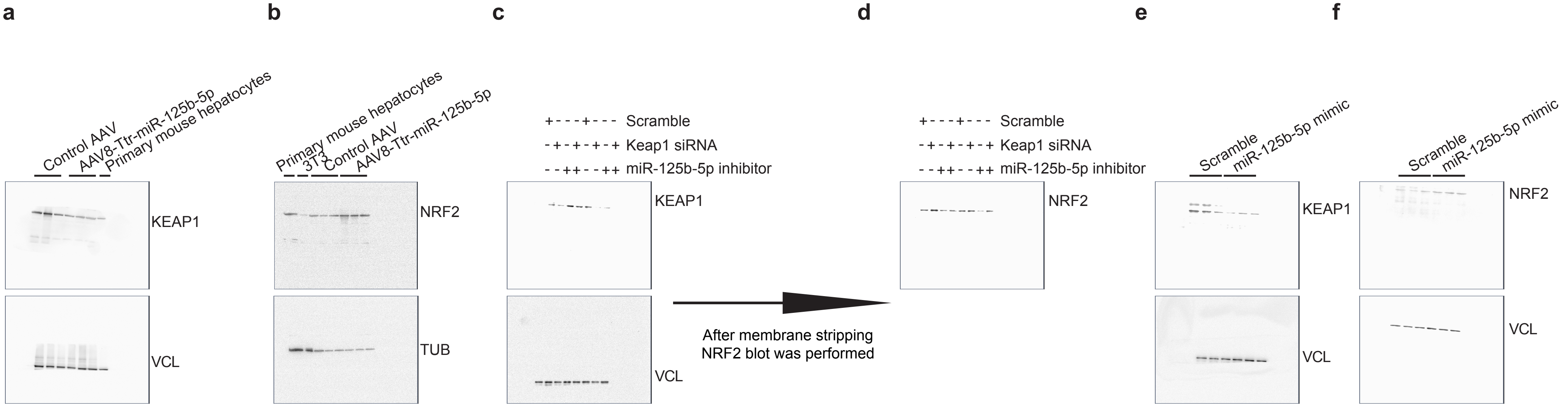

Supplementary figure 10: Scans of original blots presented in Figs. 4-6.
(a) Fig. 4b, KEAP1 (top) and vinculin (below). **(b)** Fig. 4g, NRF2 (top) and tubulin (below). **(c)** Fig. 5a, KEAP1 (top) and vinculin (below). **(d)** Fig. 5g, NRF2 (top) and vinculin blot was same as shown in panel c because NRF2 blot was performed on stripped membrane of panel c. **(e)** Fig. 6a, KEAP1 (top) and vinculin (below)**. (f)** Fig. 6g, NRF2 (top) and vinculin (below).
